# HOX cofactors expression and regulation in the human ovary

**DOI:** 10.1186/1477-7827-6-49

**Published:** 2008-10-30

**Authors:** Takayo Ota, Haruka Asahina, Se-Hyung Park, Qing Huang, Takashi Minegishi, Nelly Auersperg, Peter CK Leung

**Affiliations:** 1 Department of Obstetrics and Gynecology, the University of British Columbia (UBC), Vancouver, BC, V6H 3V5, Canada; 2 Department of Gynecology and Reproductive Medicine, Gunma University Graduate School of Medicine, Gunma, 371-8511, Japan

## Abstract

**Background:**

HOX cofactors enhance HOX binding affinities and specificities and increase HOX's unique functional activities. The expression and the regulation of HOX cofactors in human ovaries are unknown.

**Methods:**

In this study, the expression of HOX cofactors, PBX1, PBX2, and MEIS1/2, were examined by using RT-PCR, immunofluorescence in cultured immortalized human granulosa (SVOG) cells. The distribution of these HOX cofactors in human ovaries was examined by immunohistochemistry. The effects of growth differentiation factor-9 (GDF-9) and follicle-stimulating hormone (FSH) on PBX2 in SVOG cells were investigated by western blot analysis. Binding activities of HOXA7 and PBX2 to the specific sequences in granulosa cells were determined by electrophoretic mobility shift assay (EMSA).

**Results and conclusion:**

In SVOG cells, PBX1, PBX2 and MEIS1/2 were expressed during cell culture. In normal human ovaries, PBX1 and MEIS1/2 were expressed in granulosa cells at essentially all stages of follicular development. These cofactors were expressed in the nuclei of the granulosa cells from the primordial to the secondary follicles, whereas beyond multilayered follicles was observed in the cytoplasm. The co-expression of PBX1 and MEIS1/2 in granulosa cells in normal human ovaries suggested that MEIS1/2 might control PBX1 sublocalization, as seen in other systems. PBX2 was not expressed or weakly expressed in the primordial follicles. From the primary follicles to the preovulatory follicles, PBX2 expression was inconsistent and the expression was found in the granulosa cell nuclei. The PBX2 expression pattern is similar to HOXA7 expression in ovarian follicular development. Furthermore, FSH down-regulated, GDF-9 did not change PBX2 expression, but co-treatment of the granulosa cells with FSH and GDF-9 up-regulated PBX2 expression. These results implicated a role for PBX2 expression in the steroidogenic activities of granulosa cells in humans. Moreover, PBX2 and HOXA7 bound together to the Pbx sequence, but not to the EMX2 promoter sequence, in SVOG cells. Our findings indicate that HOX cofactors expression in normal human ovary is temporally and spatially specific and regulated by FSH and GDF-9 in granulosa cells. HOX proteins may use different HOX cofactors, depending on DNA sequences that are specific to the granulosa cells.

## Background

Transcription factors play important roles in oogenesis and folliculogenesis [1,2]. Numerous studies have emphasized the importance of ovarian-specific transcription factor genes during ovarian follicular development. However, non-ovarian-specific genes are also expressed during ovarian follicular development, and their potential functions in folliculogenesis, such as steroidogenesis, are not known [3-5].

Homeobox genes are transcription factors that encode homeodomain-containing DNA-binding proteins that specify the anterior-posterior orientation of a variety of organs during embryonic development and regulate differentiation in the adult tissues. The homeodomain is highly conserved in Hox proteins. Hox share similar DNA binding specificities *in vitro*. In vivo, Hox cofactors enhance their target DNA binding affinities and specificities [6,7]. Homeobox domains in Hox cofactors contain a three-amino acid loop extension (TALE), which can be classified into two groups: Pbx and Meis/Prep. Pbx binds to groups 1 to 10 (out of 13 paralogous Hox groups) and Meis proteins bind to groups 9–13. However, recent studies have shown that Meis1 also can bind to anterior Hox proteins [8] in vitro. In addition, Meis proteins interact directly with Pbx and participate in the DNA bound Hox complex and regulate the sublocalization of Pbx [7].

Pbx has four subclasses: Pbx1–4. Pbx1 was first identified in acute pre-B-cell leukemias [9]. The Pbx2, Pbx3, Pbx4 genes were subsequently identified [10,11]. In the female reproductive system, Pbx1 is expressed in the coelomic epithelium [12]. Pbx1 encodes two spliced variants Pbx1a and Pbx1b. Pbx1a is not expressed in the developing female reproductive systems, whereas Pbx1b is expressed in the Müllerian ducts at embryonic day 14.5 (E14.5) [13]. Pbx1-deficient mice die at E15.5-E16.5, with axial skeletal malformations and abnormalities of multiple organs [14], including improper differentiation of the gonads and an absence of Müllerian ducts [12]. In developing embryos, at E14.5, weak Pbx3 expression was observed throughout the ovary [15]. Pbx3-deficient mice survive to term but die within a few hours of birth [16], and there is no description about reproductive system anomalies thus far. The expression patterns of Pbx1 and Pbx3 are overlapped during embryonic development, and they could have redundant functions. In contrast, Pbx2 is not crucial for development, which implies possible compensation by other Pbx isoforms [17].

Three Meis isoforms (Meis1, Meis2 and Meis 3) are all expressed in newborn and 10-week old mice in the ovary [8]. Meis1 null mice die at E14.5 and are deficient in normal capillaries, megakaryotes and eyes [18,19], but there was no description about the reproductive system in these reports. Immunohistochemical studies have shown that, in newborn female reproductive systems, Meis1 is expressed throughout the ovary, uterus, cervix and vagina [8]. Meis2 is not expressed in the gonad primodium at E9.5–11.5, but, in adult mice, the Meis2c and Meis2d isoforms are expressed [20]. MEIS1 and MEIS2 are expressed in the nuclei of normal adult human ovarian surface epithelium; however, their functions in the ovary have not been elucidated [21].

In this study, we examined the immunohistochemical distribution of HOX cofactors in normal human premenopausal ovaries. We also examined PBX1, PBX2 and MEIS1/2 expression in immortalized granulosa cells in vitro. Furthermore, because PBX2 expression was observed to be similar to HOXA7 as seen in our previous study [3], we focused on PBX2 and investigated the regulation of PBX2 by FSH and GDF-9 treatment and on the DNA binding activities of PBX2 and HOXA7 in granulosa cells.

## Methods

### Materials

The antibodies for PBX1 (sc-889), PBX2 (sc-890), MEIS1/2 (sc-10599), MEIS1/2 blocking peptide (sc-10599p) and actin (C-11) were purchased from Santa Cruz Biotechnology Ltd. (Santa Cruz, CA). The epitopes of the MEIS1/2 antibody was designed for MEIS1, however, has 94% similarities with MEIS2. Human recombinant FSH was purchased from Dr. A.F. Parlow (National Hormone and Pituitary Program, CA). GDF-9 was kindly provided by Dr. A. J.W. Hsueh (Stanford University, CA).

### Tissues

The use of human tissues and cells was approved by the committee for ethical review of research involving human subjects of the University of British Columbia. Paraffin sections were obtained from grossly and histologically normal ovaries of 7 premenopausal women, collected by the Department of Pathology (UBC) with patient consent. Premenopausal status was confirmed by the presence of follicles or corpora lutea. Carcinoma was excluded in all of the ovarian samples used in this study by histopathological assessment.

### Cell culture

SV40 Tag-immortalized human granulosa cells (SVOG) were generated, as described previously [22], by transfection of the SV40 early genes Tag/tag into human granulosa cells obtained from IVF procedures. SVOG cells were maintained in 199/MCDB 105/10% FBS with 0.4 μg/ml hydrocortisone (BD Biosciences Clontech, Mountain View, CA). A cervical cancer cell line, Hela, and an erythroleukemic cell line, K562, were purchased from the American Type Culture Collection (ATCC). Ishikawa cells were provided by Dr. Taylor (Yale University). Hela cells were maintained in DMEM (Invitrogen, Burlington, ON) supplemented with 10% FBS (Hyclone Laboratories Ltd., Logan, UT). K562 cells were maintained in RPMI 1640 medium (Invitrogen) with 15% FBS. Ishikawa cells were maintained in DMEM/F-12 with 5% FBS. All cells were grown in a 5% CO_2_/air atmosphere, and Hela, Ishikawa and SVOG cells were passaged using 0.06% trypsin/0.01% EDTA in Ca^2+^-, Mg^2+^-free Hanks' BSS.

### RT-PCR

Total RNA was extracted from cultured cells using the RNeasy kit (Qiagen Inc., Mississauga, ON) according to the manufacturer's procedure. Complementary DNA was transcribed from 1.5 μg total RNA using a First Strand cDNA synthesis kit (Amersham, Oakville, Ont., Canada) and used as a template for polymerase chain reaction (PCR). All PCR primers span introns in order to detect specific mRNA sequences. The forward and reverse primers used were as follows: PBX1 (NM_002585): 5'-CCACGTGATGAATCTCCTGCGAGAG-3' and 5'-TCACTGTATCCTCCTGTCTGGCTGA-3', PBX2 (NM_002586): 5'-CTGGTTTGGCAACAAGAGGATTCGC-3' and 5'-TGGAGGTATCAGAGTGAACACTCCC-3' and MEIS1 (BC043503): 5'-AAGGTGATGGCTTGGACAA-3' and 5'-GGCTGCACTATTCTTCTCCG-3'. The expected size of PCR products using these sets of primers are 627 bp for PBX1a, 414 bp for PBX1b, 411 bp for PBX2, and 259 bp for MEIS1. The amplification reaction was carried out in the linear range of the logarithmic phase unless otherwise specified. Each cycle consisted of denaturation at 95°C for 30 s, primer annealing at 55°C for 30 s, extension at 72°C for 60 s and a final extension at 72°C for 5 min in a DNA thermal cycler (Mini cyclerTM, PTC-100 TM, MJ-Research, Bio-Rad). PCR products were verified by sequence analysis.

### Western blot analysis

Cells were washed with ice-cold PBS and lysed by cold lysis buffer (1% Triton X-100, 0.5% sodium deoxycholate, 0.1% SDS in PBS, pH 7.4), including freshly added protease inhibitor (Sigma, St. Lois, MO). The extracts were placed on ice for 10 min, vortexed briefly and centrifuged for 15 min at 4°C to remove cell debris. The total protein concentration was determined using a Bradford assay (Bio-Rad Laboratories, Mississauga, ON). The samples were boiled for 10 min before running the gels. SDS polyacrylamide gel electrophoresis (SDS-PAGE) was performed using a 10% separating gel. Twenty-five to 30 μg of protein was then electrotransferred to a nitrocellulose membrane (Bio-Rad Laboratories). The membrane was blocked with 5% skim milk in PBS. PBX2 and actin were detected by primary antibodies in 5% skim milk/TBS for 1 hr at 25°C. Subsequently, the signals were detected with secondary antibodies conjugated with horse radish peroxidase and visualized by ECL (Pierce, Rockford, IL).

### Immunofluorescence

For PBX1 and MEIS1/2, cells were grown on glass coverslips, fixed in freshly prepared 4% paraformaldehyde in PBS for 10 min at 4°C, and permeabilized with 0.1% Triton X-100 in PBS for 10 min at 25°C. For PBX2, cells on the coverslips were fixed in cold methanol and postfixed in cold methanol/acetone (1:1). The cells were incubated with primary antibodies, anti-PBX1 (1:20), anti-PBX2 (1:50) and anti-MEIS1/2 antibody (1:50). The binding of primary antibodies was followed by goat anti-mouse antibody (Alexa Fluor 594, Molecular Probes, Eugene, Oregon) or rabbit anti-goat antibody (Alexa Fluor 488, Molecular Probes). Control experiments were done in the absence of primary antibodies and verified their no or little background. For Hoechst staining, after incubation of the secondary antibodies, cells were incubated for 1 min with 5 μg/ml Hoechst 33258.

### Immunohistochemistry

Ishikawa, Hela and K562 cell pellets were solidified by collagen gel, fixed in 4% paraformaldehyde, and embedded in paraffin. Sections of these cells and of the ovaries were deparaffinized, rehydrated, and submitted to antigen retrieval by a steamer for 30 min in 10 mM citric buffer (PBX2) or Target Retrieval Solution (DAKO, Mississauga, ON; PBX1 and MEIS1/2). Endogenous peroxide was diminished with 3% H_2_O_2 _for 30 min. Nonspecific binding was blocked with 3% BSA in PBS (PBX2) or Protein Block (DAKO; PBX1 and MEIS1/2) for 30 min, followed by incubation with primary antibodies overnight at 4°C. The dilution of the primary antibodies was the same as that described for the immunofluorescence. Samples without primary antibodies were used as a negative control. There was no or little background without primary antibodies. The sections were then incubated with Biotinylated link universal (DAKO) for 15 min and streptavidin (DAKO) for 25 min at 25°C. Slides were developed in diaminobenzine (DAKO) or in NovaRED substrate (Vector Laboratories, Inc., Burlington, ON) and counterstained with hematoxylin.

### Electrophoretic Mobility-Shift Assay (EMSA)

Nuclear extracts were prepared with a nuclear extract kit (Panomics, Inc., Redwood City, CA) according to the manufacturer's instructions. DNA-binding reactions were performed with equal amounts of nuclear proteins (10 μg) and ^32^P-labeled probes at 25°C for 10 min. The following synthetic double-strand oligonucleotide probes were used: Pbx, 5'-CGAATTGATTGATGCACTAATTGGAG-3', and EMX2, 5'-AGGAAGCTGTTTATGTGATCCCCG-3', which have been previously shown to contain the consensus recognition sequences for the HOX-Pbx complexes [23,24]. For cold competition assays, a 100-fold excess of unradiolabeled oligonucleotides was added to the reactions prior to the ^32^P-labeled probes. Anti-HOXA7 and anti-PBX2 antibodies used in the supershift experiments were added to the nuclear extract at 25°C for 30 min before the addition of labeled probe. Protein-DNA complexes were resolved in 5% polyacrylamide gel containing 1× TBE (Tris-borate-EDTA: 0.09 M Tris-borate and 2 mM EDTA, pH 8.0). Before loading of the samples, the gel was pre-run for 90 min at 100 V at 4°C. Electrophoresis was carried out at 120 V at 4°C. The gel was then dried under vacuum and exposed to Kodak X-OMAT AR film (Kodak, Rochester, NY).

## Results

### Expression of HOX cofactors in immortalized granulosa cells

**Figure 1:**
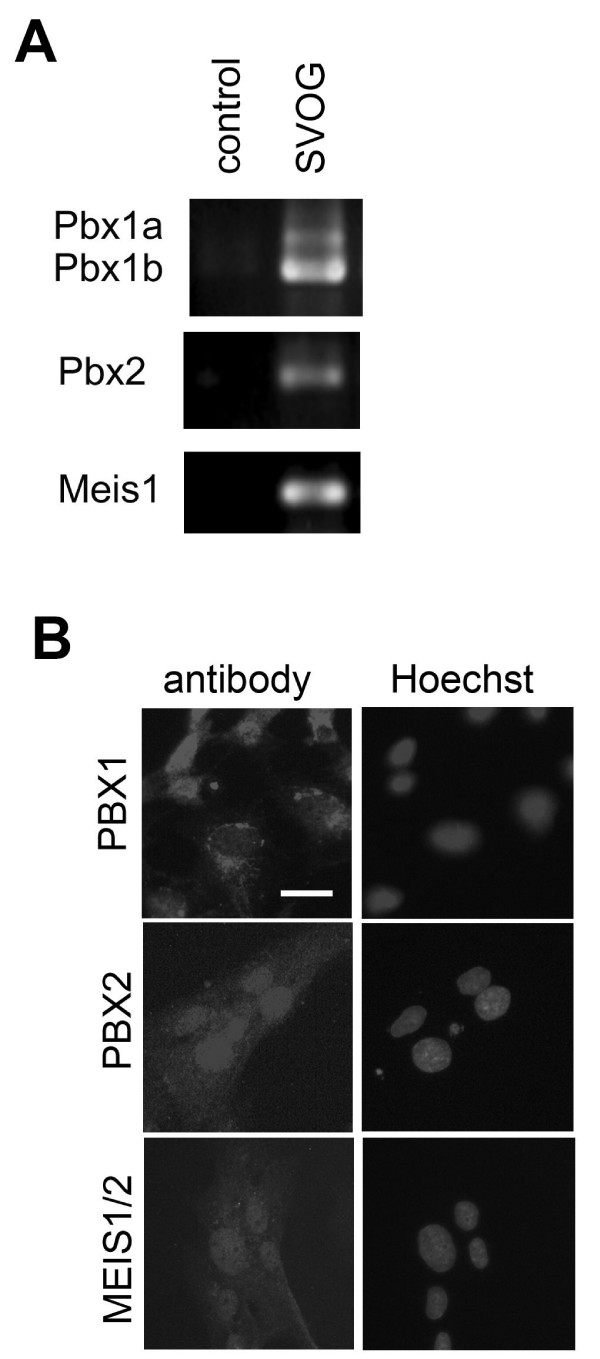
**Expression of HOX cofactors in immortalized human granulosa (SVOG) cells in culture**. (a) mRNA levels of PBX1, PBX2 and MEIS1. control, no cDNA. (b). Levels of PBX1, PBX2 and MEIS1/2 as shown by immunofluorescence microscopy. The staining of nuclei with Hoechst is shown in the right column. (scale bar = 20 μm).

Recent studies have identified a number of HOX genes in the oocytes, and we have previously shown the distribution of HOXA7 in ovarian follicular development [3]. To examine the expression of HOX cofactors in normal human ovary, we focused on PBX1, PBX2 and MEIS1/2. In addition, to determine the expression of HOX cofactors in human granulosa cells, we performed RT-PCR following the culture of SVOG cells. Figure 1A showed that PBX1, PBX2 and MEIS1 are transcribed in proliferating SVOG cells. Two transcripts corresponding to PBX1a and PBX1b were detected, and PBX1b, which lacks the C-terminal domain in PBX1a, was the more predominant form in SVOG cells (Figure 1A). The specificities of the HOX cofactors antibodies were confirmed by using paraffin-embedded Ishikawa, Hela and K562 cells (data not shown). By immunofluorescence, PBX1 was observed to be expressed both in the nuclei and in the cytoplasm, which is consistent with the results reported in Hela or Ishikawa cells (Figure 1B) [25]. In SVOG cells, PBX2 and MEIS1/2 were expressed in the nuclei.

### Immunohistochemistry

**Figure 2:**
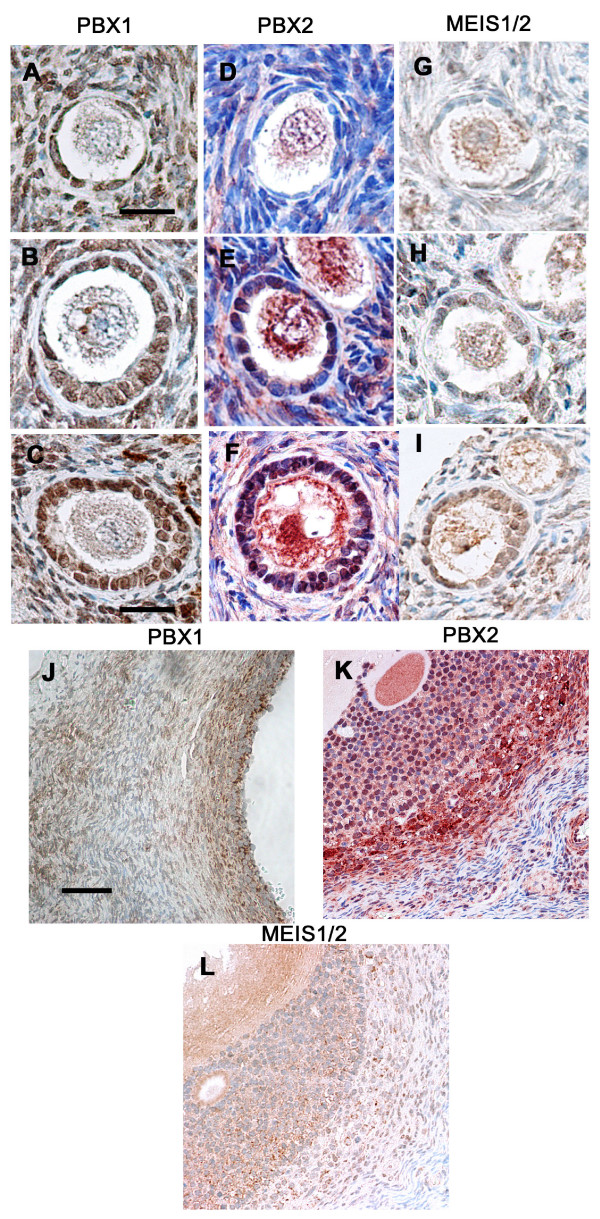
**Immunohistochemical staining for PBX1, PBX2 and MEIS1/2 in human ovaries**. (A, B, C, J) PBX1, (D, E, F, K) PBX2 and (G, H, I, L) MEIS1/2. (A, D, G): primordial follicles. (B, E, H): primary follicles. (C, F, I): secondary follicles. (J, K, L): preovulatory follicles. Figure 2E contains both primordial and primary follicles, and Figure 2I contains both primordial and secondary follicles (scale bars: A,B,D,E,G,H = 9 μm, C,F,I = 40 μm, J,K,L = 350 μm).

The distribution of PBX1, PBX2 and MEIS1/2 was examined by immunohistochemistry on paraffin sections of premenopausal ovaries. Five ovaries were used for PBX1 and 6 ovaries were used for MEIS1/2 and PBX2. Immunohistochemical specificity of the anti-MEIS1/2 antibody was confirmed by using MEIS1/2 blocking peptide (data not shown). As shown in Figure 2, oocyte nuclei were negative, and oocyte cytoplasms were weakly positive, at all stages for PBX1, PBX2 and MEIS1/2. PBX1 staining was observed in the nuclei of granulosa cells from primordial to secondary follicles. In 50% of primordial follicles, granulosa cells were partially positive (Figure 2A; Table 1). In contrast, in primary and secondary follicles, almost all granulosa cells were completely positive (Figure 2B, C; Table 1). In multilayered and Graffian follicles, similar to HOXA7, PBX1 had mainly translocated to the cytoplasm (Figure 2J). The theca interna were positive, and the theca externa were weakly positive (Figure 2J). In the theca, the expression was also in the cytoplasm. MEIS1/2 expression was quite similar to PBX1 expression (Figure 2G, H, I, L). In primordial follicles, the percentage of MEIS1/2 negative granulosa cells was more compared to PBX1 (Table 1). Other differences between PBX1 and MEIS1/2 stainings were that the theca interna were positive but showed both nuclear and cytoplasmic staining for MEIS1/2 (Figure 2L).

**Table 1:** Expression of HOX cofactors in granulosa cells during follicular development

	negative	positive	Strongly positive	total
**PBX1**				
primordial	1 (6%)	9 (50%)	8 (44%)	18
primary	1 (2.5%)	1 (2.5%)	41 (95%)	43
secondary	0 (0%)	0 (0%)	2 (100%)	2
multi, Graffian	4 (100%)	0 (0%)	0 (0%)	4
**PBX2**				
primordial	4 (21%)	15 (79%)	0 (0%)	19
primary	3 (10%)	20 (67%)	7 (23%)	30
secondary, multi, Graffian	0 (0%)	5 (83%)	1 (17%)	6
**MEIS1/2**				
primordial	8 (36%)	12 (55%)	2 (9%)	22
primary	6 (18%)	27 (79%)	1 (3%)	34
secondary	0 (0%)	0 (0%)	4 (100%)	4
multi, Graffian	5 (100%)	0 (0%)	0 (0%)	5

PBX2 expression in normal human ovaries was completely different from PBX1 and MEIS1/2 expression. Interestingly, the expression pattern was similar to HOXA7 expression as we previously observed [3]. Granulosa cells in primordial follicles were negative or weakly positive (Figure 2D; Table 1). From the primary to Graffian follicles, the intensity of PBX2 expression in granulosa cells became higher when compared to the primordial follicles and the expression pattern was mixed, i.e., negative and positive (Figure 2E, F, K; Table 1). The cells of the theca interna were positive, and the cells of the theca externa were negative, which was again similar to HOXA7 expression. PBX2 expression in the theca interna was in the nuclei.

### Effects of FSH and GDF-9 on PBX2 expression

**Figure 3:**
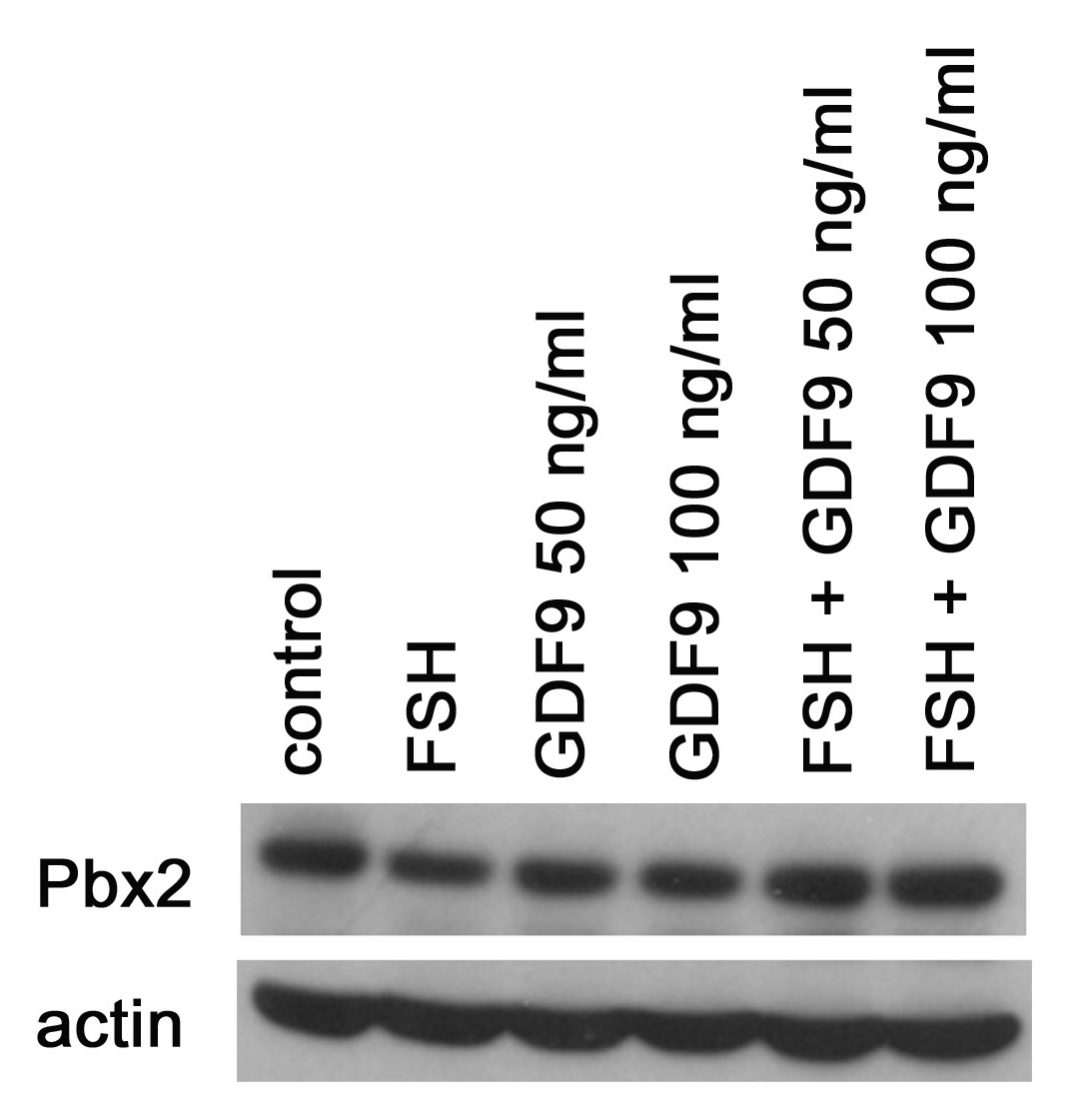
**Effects of FSH and GDF-9 on PBX2 in SVOG cells**. The cells were treated with FSH (100 ng/ml) or/and GDF-9 (50 or 100 mg/ml). For co-treatment, cells were pre-treated with FSH for 4 hr and then treated with GDF-9 for 24 hr.

Since PBX2 expression in human normal ovaries was observed to be similar to HOXA7 expression, we focused on the regulation of PBX2 expression. In our previous study, HOXA7 is regulated by GDF-9 [3]. In the present study, to investigate whether PBX2 expression is regulated in SVOG cells, the cells were treated with FSH (100 ng/ml) and/or GDF-9 (50 or 100 ng/ml). Our results showed that PBX2 expression was down-regulated by treatment with FSH, but not changed by GDF-9 alone (Figure 3). Interestingly, concomitant treatment with FSH and GDF-9 up-regulated the expression of PBX2 when compared to the control (Figure 3).

**Figure 4:**
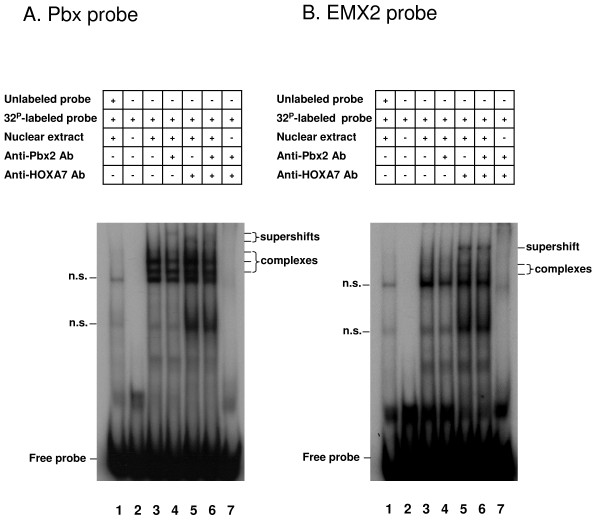
**HOXA7 and PBX2 bind as a heterodimer to the Pbx sequence, but not to EMX2**. EMSA analysis was performed with ^32^P-labeled oligonucleotides containing the (A) Pbx consensus sequence and (B) EMX2 consensus sequence. Supershift assays were also performed with antibodies to HOXA7 and PBX2. Lanes are designated as follows: lane 1, negative control using excess unlabeled cold probe (100×) with nuclear extract; lane 2, ^32^P-labeled probe without nuclear extract, showing the migration of free probe in absence of nuclear extract; lane 3, ^32^P-labeled probe with nuclear extract; lane 4, supershift reaction with ^32^P-labeled probe, nuclear extract and anti-PBX2 antibody; lane 5, supershift reaction with ^32^P-labeled probe, nuclear extract and anti-HOXA7 antibody; lane 6, supershift reaction with ^32^P-labeled probe, nuclear extract, anti-Pbx2 and anti-HOXA7 antibodies; lane 7, ^32^P-labeled probe without nuclear extract but with anti-PBX2 and anti-HOXA7 antibodies. Abbreviation: n.s., non-specific complex.

HOXA7 and PBX2 bind to the Pbx consensus sequence as a heterodimer The HOX-PBX heterodimeric complex can bind to the Pbx and EMX2 consensus sequences [23,24,26]. Therefore, we investigated the possibility that HOXA7 and PBX2 may cooperatively bind to these two DNA sequences by EMSA. PBX and EMX2 binding activities were observed in SVOG cells (Lane 3, Figure 4A, B). The specificity of binding to the respective probes was determined by using a 100-fold molar excess of unlabeled oligonucleotide as a competitor, which indicated that the bound complexes resulted from sequence specific DNA-protein interactions (Lane 1, Figure 4A, B).

To examine whether HOXA7 and PBX2 were present in the complexes, supershift analysis was performed using antibodies directed against the two proteins. Strikingly, the addition of anti-HOXA7 and/or anti-PBX2 antibodies caused supershifts of the complexes bound to the Pbx sequence, resulting in two bands with apparently higher molecular weights (Lanes 4, 5 and 6, Figure 4A). In contrast, whereas addition of the anti-HOXA7 antibody produced a characteristic shift in the complexes formed with the EMX2 probe, the PBX2 antibody alone did not result in a marked supershift (Lanes 4, 5, Figure 4B). These data revealed a differential recruitment of PBX2 to the Pbx and EMX2 consensus sequences. HOXA7 may bind to Pbx as a heterodimer with PBX2 and to EMX2 with other cofactor(s).

## Discussion

In this study, we demonstrated for the first time the expression of HOX cofactors in normal human ovaries and, particularly, in human granulosa cells. During cell culture, PBX1 was observed in the nuclei and the cytoplasm, whereas PBX2 and MEIS1/2 were observed only in the nuclei in proliferating SVOG cells. In normal human ovaries in situ, PBX1 and MEIS1/2 were expressed in the nuclei in granulosa cells from primordial to secondary follicles. In Graffian follicles, the expression of both PBX1 and MEIS1/2 was translocated from the nuclei to the cytoplasm of the granulosa cells. There is a correlation between PBX1 expression and the cell cycle [25]. PBX1 expression in the nucleus from the primordial to the secondary follicles might show that granulosa cells are at the G2/M phase, while the cytoplasmic PBX1 expression in preovulatory follicles might indicate that these granulosa cells are not dividing. Compared to ovaries in mice, PBX1 expression in human ovaries was more evident, and PBX2 expression was similar [5]. The discrepancies might be due to species differences. MEIS1 and MEIS2 control PBX1 nuclear localization where the PBX1 gene is functional [27], whereas recent studies have shown that PBX1 nuclear localization does not require both MEIS proteins [25]. In our in vitro experiments, MEIS1/2 was consistently observed in the nuclei of SVOG cells, while PBX1 was expressed both in the nuclei and in the cytoplasm. In contrast, during ovarian follicular development, PBX1 and MEIS1/2 were expressed in the same pattern in situ, which indicates that MEIS1/2 might control PBX1 nuclear localization in vivo. The explanation underlying the subcellular localization differences in PBX1 and MEIS1/2 in vitro and in vivo is not known.

Similar to HOXA7, PBX2 expression was negative in primordial follicles but positive from the stage of primary follicles. Furthermore, the mixed negative and positive expression pattern was also similar to HOXA7. One difference is in preovulatory follicles; PBX2 was expressed in the nuclei, whereas HOXA7 is expressed mainly in the cytoplasm [3]. Thus far, the role of different sublocalization of HOXA7 is not known. These results support the notion that HOXA7 and PBX2 might act together in granulosa cells, especially when they are co-expressed in the nuclei.

By FSH and/or GDF-9 treatment, PBX2 expression in granulosa cells might be reversely related to steroidogenic activities. The immortalized granulosa (SVOG) cells used in this study are steroidogenic, as demonstrated by increased progesterone production following treatment with cAMP or pregnenolone in our earlier report [22]. It is well known that FSH and cAMP up-regulates StAR expression and stimulates steroidogenesis in human granulosa cells [28]. In the present study, treatment of SVOG cells with FSH alone decreased PBX2 expression. GDF-9 on its own did not change StAR [28], and, in our study, GDF-9 had no or weak inhibitory effects on progesterone production in SVOG cells (data not shown), and it did not change PBX2 expression. FSH or cAMP-stimulated progesterone production, which is mediated through StAR, was potently inhibited by GDF-9 co-application in human granulosa cells [28]. In the present study, PBX2 expression was increased by the co-application of FSH and GDF-9. Hence, PBX2 expression might be inversely related to steroidogenesis in human granulosa cells. This notion is in accordance with our demonstration that PBX2 was expressed in the granulosa and theca interna cells that produce estrogen and androgen, respectively, but not in theca externa that lack steroidogenic activity.

To validate the PBX2 and HOXA7 protein-protein interaction in SVOG cells, we examined their binding to two target sequences: PBX and EMX2. Previous studies showed that complexes of Pbx1 and HOX1–4 display optimal binding to the target sequence 5'-CGAAT**TGAT**TGATGCAC**TAAT**TGGAG-3' [23] and Pbx2 is also known to bind to this sequence [26]. TGAT is a Pbx binding site and TNAT is a HOX site. The TAAT, the HOX binding site which was used in this study, is accepted to bind the middle paralog groups 3–8 [29]. In addition, PBX2 and HOXA10 interactions with EMX2 were demonstrated in endometrial cancer cell lines [24]. EMX2 is expressed in the epithelial components of the urogenital system during development, and, as shown by its knockout studies, this gene is essential for the development of the female reproductive system [30]. In this study, HOXA7 and PBX2 complexes bound to the Pbx sequence, but not to the EMX2 sequence. The results indicate that a HOXA7 and PBX2 interaction occurs in granulosa cells. EMX2 can be a target of HOXA7, but it did not bind to PBX2 in granulosa cells. These results suggest that HOXA7 and PBX2 can make dimers in granulosa cells. However, when HOXA7 binds to the EMX2 promoter in granulosa cells, different cofactors might be used to enhance the HOXA7 binding specificity and strength.

## Conclusion

In summary, this study demonstrated, for the first time, the expression and regulation of HOX cofactors in normal human ovaries. The results indicate that PBX1 and MEIS1/2 might act together and that PBX2 and HOXA7 act together at specific promoter regions in human granulosa cells. It appears that PBX2 expression is closely related to steroidogenic activities in granulosa cells and that it plays a role in ovarian follicular development.

## Competing interests

The authors declare that they have no competing interests.

## Authors' contributions

TO and HA designed the study and performed the molecular genetic studies, the immuno-technical studies and participated in discussion of the results and drafted the manuscript. SP performed the EMSA and QH performed immunoassays and both of them participated in discussion of the results and critical revision of the manuscript. TM, NA and PCKL were responsible for supervision of this work. PCKL was responsible for the conception, design, discussion of the results, drafting and critical revision of the manuscript. All authors read and approved the final manuscript.
